# Classification and Assessment of Medication Risk in the Elderly (CARE): Use of a Medication Risk Score to Inform Patients’ Readmission Likelihood after Hospital Discharge

**DOI:** 10.3390/jcm10173947

**Published:** 2021-08-31

**Authors:** Savanna SanFilippo, Veronique Michaud, Juanqin Wei, Ravil Bikmetov, Jacques Turgeon, Luigi Brunetti

**Affiliations:** 1 Robert Wood Johnson University Hospital Somerset, 110 Rehill Avenue, Somerville, NJ 08876, USA; savanna.sanfilippo@rwjbh.org (S.S.); juanqin.wei@rwjbh.org (J.W.); 2 Tabula Rasa Health Care, 13845 Veteran’s Way Suite 410, Lake Nona, FL 32827, USA; vmichaud@trhc.com (V.M.); rbikmetov@trhc.com (R.B.); jturgeon@trhc.com (J.T.); 3 Ernest Mario School of Pharmacy, Rutgers, The State University of New Jersey, 160 Frelinghuysen Road, Piscataway, NJ 08854, USA

**Keywords:** medication, risk score, polypharmacy

## Abstract

Existing risk tools that identify patients at high risk of medication-related iatrogenesis are not sufficient to holistically evaluate a patient’s entire medication regimen. This study used a novel medication risk score (MRS) which holistically evaluates medication regimens and provides actionable solutions. The main purpose of this study was to quantify adults ≥ 65 years with a high medication risk burden using the MRS and secondarily, appraise MRS association with hospital readmission. This retrospective cohort study included all consecutive patients in a 6-month period aged 65 years and older, admitted for at least 48 h, and prescribed at least five medications upon discharge. Out of 3017 patients screened, 1386 met all criteria. The primary outcome was the proportion of patients with a score of ≥20 and the secondary outcome was the 30-day readmission rate. In the overall population, 17% of patients had an MRS ≥ 20. For patients discharged home, there was a 19% readmission rate for a score ≥ 20 and 11% for <20 (*p* = 0.009). A score of ;≥20 was associated with a 1.8-fold increased risk of readmission in patients discharged home. Only 7% of patients met these criteria, which can help direct future use of the MRS at patients with the highest risk of medication-related iatrogenesis.

## 1. Introduction

Older adults—patients over 65 years, who make up 40% of all inpatients—are particularly susceptible to medication-related iatrogenesis and 30-day readmission [1]. Approximately 20% of Medicare beneficiaries, who are ≥65 years with few exceptions, are readmitted to a hospital within 30 days, compared to the national average of 15.7% [2]. When older adults are readmitted within 30 days, there is an increased risk of 1-year mortality, a dramatic increase in the risk of repeated 30-day readmissions, and increased overall healthcare costs [3,4]. Approximately 13% of avoidable readmissions are due to adverse drug events, almost all of which are serious and preventable [4]. A tool for the accurate assessment and mitigation of medication-related iatrogenesis risk upon discharge from the acute care setting is needed to reduce 30-day readmissions and improve patient outcomes.

Existing measures of medication risk are not sufficient to holistically evaluate medication regimens. Commonly used tools to predict risk specifically for elderly patients include the PADR-EC score, BADRI model, and GerontoNet ADR risk [3,5,6,7]. The PADR-EC score, for example, is used to predict hospitalization due to adverse drug events (ADEs) in outpatients of 65 years and older [5]. These existing risk rating tools are based on patient-specific factors or simply the number of medications in a specific therapeutic category. Moreover, many are geared to identify patients at high risk of medication-related iatrogenesis in specific, rather than all, categories. They often focus on single therapeutic categories, address mainly nonmodifiable factors, or have only outpatient validation. There are countless scoring tools for the anticholinergic burden, such as the Anticholinergic Activity Scale, Anticholinergic Burden Classification, and Anticholinergic Drug Scale [8,9,10]. These focus on specific medications but are cumbersome to calculate by hand. Similarly, the Michigan Opioid Safety Score and Opioid Risk Tool are more focused on the risk of sedation or abuse, rather than the overall aggregated risk a medication regimen poses to a patient [11,12].

The proprietary advanced clinical decision support system and medication risk score used in this study uses the FDA Adverse Event Reporting System (FAERS), the Anticholinergic Cognitive Burden Scale, sedative burden, QTc prolongation risk measures, and CYP450 drug interaction burden. MRS calculation details have been previously published [13,14,15].

The resulting score ranges from 0–53; a score of less than 10, 10–14, 15–19, 20–30, and greater than 30 is interpreted as minimal, low, intermediate, high, and severe risk, respectively [16]. The tool was validated in a cohort of 1965 patients of the Programs of All-Inclusive Care for the Elderly. This study found a one-point increase in the score correlates with an 8.6% odds increase of one or more ADEs per year, $1000 in annual medical spending, and 2.1 additional hospitalizations per year [17]. This score was also demonstrated to be associated with an increased risk of premature death in more than 400,000 patients followed for 7 years [16]. In addition to a medication risk score, the tool provides clinical decision support and consequently provides actionable ways to reduce medication risk. The multifactorial approach and actionable methods to reduce the risk score differentiate this tool from others used in the inpatient setting. However, as with other risk tools, inpatient validation is required and cannot necessarily be inferred from outpatient studies. The purpose of this study is to quantify the total medication risk burden in elderly patients at discharge from the acute care setting and to appraise the association of this novel medication risk score with hospital readmission.

## 2. Materials and Methods

### 2.1. Study Design

We performed a single-center retrospective cohort study of all consecutive patients meeting the inclusion and exclusion criteria at a large community teaching hospital. The aim was to evaluate the influence of medications on hospital readmission within 30 days. The medication risk score, the MedWise Risk Score^TM^ (MRS), was calculated for all patients using an algorithm combining the following: risk of ADE using the FDA Adverse Event Reporting System (FAERS), Anticholinergic Cognitive Burden, Sedative Burden, QTc prolongation risk, and CYP450 drug interaction burden [13,14,15]. Microsoft SQL Server (v.15) was used to manipulate and analyze data to generate the MRS. The follow-up period included 30 days post discharge from the index admission. The study was granted approval by the Robert Wood Johnson University Hospital Somerset Institutional Review Board (IRB 20–31). A waiver of consent was granted because of the study’s retrospective nature and lack of intervention.

### 2.2. Patient Selection

Data for all consecutive patients aged 65 or greater hospitalized for 48 h or more were extracted directly from the electronic medical record (EMR) system, Allscripts Sunrise Clinical Manager. To be eligible for inclusion, patients needed to be prescribed at least five medications upon discharge. All medications, including inhalers, over the counter, supplements and herbals, topicals, and those scheduled as needed were included in the number of medications in the patient’s regimen. Patients who resided more than 25 miles from the hospital, were transferred to another acute care facility, were discharged against medical advice, or expired during index hospitalization were excluded from the study in an effort to remove patients who may seek medical care at other hospitals. If patients had multiple admissions in the 6-month study period or had multiple readmissions, only the first admission and first readmission were included.

### 2.3. Measurements

All relevant admission data, including demographics (age, sex, race, etc.), diagnosis codes, and discharge medications were collected from the EMR. The age of patients over the age of 89 was censored to 89 years to comply with the Health Insurance Portability and Accountability Act (HIPAA). International Classification of Diseases, Tenth Revision, Clinical Modification (ICD-10-CM) codes were used to identify comorbidities and to calculate the age-adjusted Charlson Comorbidity Index to estimate 10-year survival for each patient [18]. To calculate the MRS, all medications, including strength, frequency, time of day, and directions are required. Optional factors included in the score as available were pharmacogenomic data and lab values.

### 2.4. Outcomes

The primary objective of the study was to establish the level of medication-related risk based on MRS in an elderly acute care population. The primary outcome assessed was the proportion of patients with a high or severe MRS, defined as a score greater than or equal to 20 (referred to as the high-risk group herein). Patients were then stratified into either the high-risk or control group based on the MRS (MRS ≥ 20 and MRS < 20, respectively). The secondary outcome was 30-day hospital readmissions. Prespecified subgroup analyses in patients discharged to home and to other facilities after their index admission were performed.

### 2.5. Statistical Analysis

All data were summarized using descriptive statistics. Categorical data and continuous data were presented as proportions and means, respectively. Normality of data was assessed by visual inspection of the histogram and the Kruskal-Wallis test. A Z-test was used to compare categorical data and the independent sample t-test was used to assess continuous data, both with a *p*-value threshold of <0.05. A multivariable logistic regression was constructed for 30-day readmissions using MRS as the exposure variable and 30-day readmission as the outcome variable. Confounders were selected based on prior knowledge as well as a bivariate analysis. Covariates with *p* < 0.1 in the univariate analysis were further tested in the multivariable model. Only covariates with a *p* < 0.05 were retained in the final multivariable model. All data were analyzed with SPSS version 26.0 (IBM Corporation, Armonk, NY, USA).

## 3. Results

### 3.1. Patient Selection and Demographics

**Figure 1:**
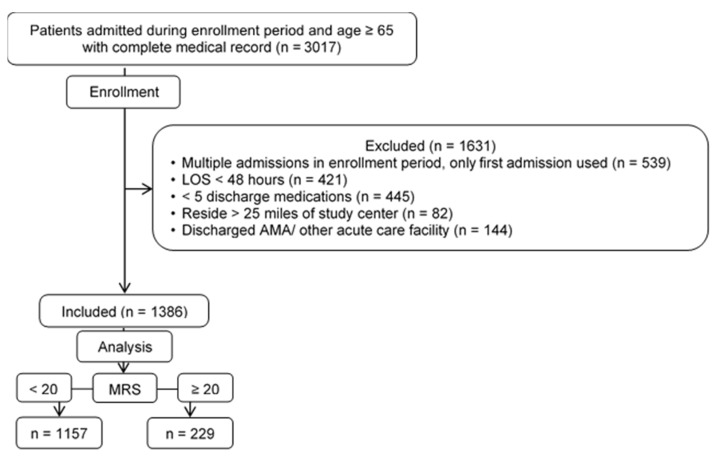
Patient Selection Process. LOS = length of stay.

A total of 3017 patients with available data were admitted between 1 July 2020 and 31 December 2020 and screened for inclusion. After application of inclusion and exclusion criteria, 1386 patient records remained and were included in the study. A summary of the patient selection process may be found in Figure 1.

Patient demographics and characteristics are provided in Table 1. Each patient, on average, was prescribed 11 medications and had a length of stay of 6 days. The readmission rate was 14% with an average time to readmission of about 2 weeks. More than half of the patients had a Charlson Comorbidity Index of four or greater, which is based solely on diagnosed disease states, indicating a 53% or less estimated 10-year survival [18,19]. Reported comorbidities occurred in 10% or more of the patients and were identified using ICD-10 codes. Additionally, there was a higher incidence of dementia among those discharged to facilities, which is to be expected due to the increased level of care required for these patients. The three most common admitting diagnoses—with incidences ranging from 5% to 9%—were heart failure, sepsis, and cardiovascular disease. For readmission, the three most common diagnoses were sepsis, hypertension, and heart failure; each causing about 10% of readmissions.

### 3.2. Outcomes

**Figure 2:**
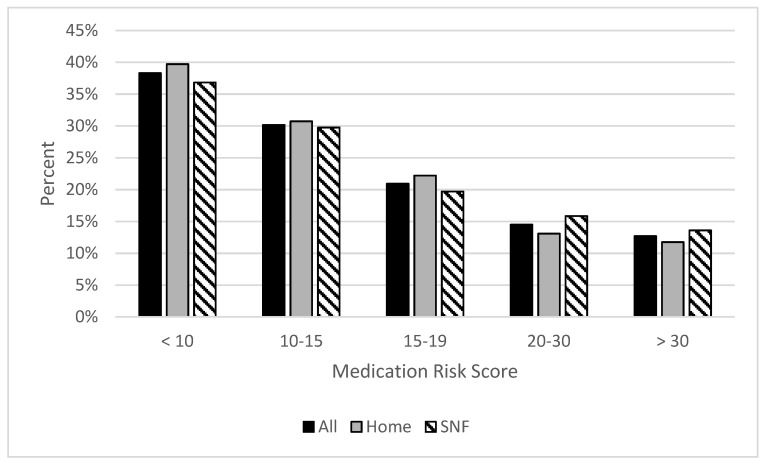
MRS Distribution of All Patients and Subgroups.

Upon stratification according to the MRS, 1157 patients were assigned to the control group and 229 patients were assigned to the high-risk group. More detailed information on score stratification can be found in Figure 2. The difference in 30-day readmissions between the high-risk and control group did not reach statistical significance (16% versus 13%, respectively; *p* = 0.412). Comparison of characteristics and outcomes of all patients between both groups can be found in Table 2.

Subgroup analyses were performed for all patients discharged home and those discharged to other facilities (including long-term care facilities and rehabilitation centers). Of the 680 patients discharged home, 582 patients were in the control group and 98 in the high-risk group. There was a statistically significant increase in 30-day readmissions in the high-risk home group versus the control (19% versus 10%, respectively; *p* = 0.009). There was a greater length of stay, proportion of patients with a Charlson Comorbidity Index > 4, and number of discharge medications in the high-risk home group compared to the home control. Although more patients in the high-risk home group had COPD, heart failure, or dementia, there was no significant difference in the readmission rate for these disease states. In the other facility group, similar results were seen, without a meaningful difference in the comorbidities reported. There was no statistically significant difference in 30-day readmissions in this group. Between the patients discharged to other facilities and home, other facility patients were more likely to be older, female, have a higher Charlson Comorbidity Index, and more medications prescribed. A summary of all patient characteristics and outcomes between groups is provided in Table 2.

To adjust for potential confounders a multivariable logistic regression was constructed. In the overall population and the discharge to other facility group, the high-risk group was not associated with an increased 30-day readmission even after adjusting for potential confounders (OR, 1.06; 95% CI, 0.71 to 1.58; *p* = 0.787) and (OR, 0.67; 95% CI, 0.38 to 1.19; *p* = 0.171), respectively. However, in the prespecified subgroup analysis including patients discharged home, the high-risk group was associated with a 1.8 times greater risk of readmission within 30 days of hospital discharge (OR, 1.81; 95% CI, 1.02 to 3.21; *p* = 0.042) (Table 3).

## 4. Discussion

Approximately 15–20% of patients were considered high-risk based on the MRS (19.8% overall and 16.8% in the discharge to home cohort). Our prespecified subgroup analysis of patients discharged home found that those deemed high-risk were 80% more likely to be readmitted, with adjustment for confounding. This finding is significant because it suggests that using the MRS to identify high-risk patients may streamline strategies, such as targeted pharmacist interventions or discharge counseling, aimed at reducing hospital readmission. Several studies have found increased age to be a risk factor specifically for ADE-related readmission; however, prioritizing patients using the MRS with a focus on those discharged home may prove more successful at reducing readmission [20,21]. While only 7% of the study’s patients fit these criteria, this small subset is ideal for risk measurement because it allows efforts to be directed toward patients at highest risk for medication related iatrogenesis and adverse outcomes.

However, the secondary outcome of the 30-day readmission rate in the overall population did not reach statistical significance (13% versus 16%, respectively; *p* = 0.412). This finding is not surprising. The overall population included patients discharged to other facilities such as long-term care facilities, rehabilitation centers, and nursing homes, which have incentives to minimize medication-related problems. The Centers for Medicare & Medicaid Services (CMS) requires “each resident’s drug regimen must be free from unnecessary drugs” and that each facility must have medication error rates of less than 5% [22]. To achieve these goals, CMS also requires facilities to implement care plans within 48 h of resident admission, have a pharmacist perform monthly drug regimen reviews, have an Antibiotic Stewardship Program, and perform biweekly reviews for patients on as-needed psychotropic medications [23]. As such, these facilities may have nursing care, consultant pharmacist reviews, and a structured formulary to reduce the potential for drug incompatibilities. As a result, they may have reduced readmissions in patients assigned to facilities upon discharge. Conversely, patients discharged home are not likely to have their medication regimens evaluated as intensely or continuously as in facilities with nursing or similar care. These nuances may also explain the lack of significance in the skilled nursing facility group despite a higher average CCI and number of medications prescribed.

ADEs are known to lead to prolonged hospital stays, temporary or permanent disability, and death [17]. Previously defined risk factors for ADEs in the acute care setting include female sex, multiple comorbidities, and polypharmacy [24]. Our findings are consistent with these data. Our high-risk groups had an average of 10% more females than in the control groups, greater CCI scores, and considerably greater numbers of medications. The lack of difference in disease-specific readmission rates (such as for COPD, diabetes mellitus, and heart failure) could indicate a potential benefit irrespective of disease state.

While the study provided compelling evidence that the MRS is beneficial when used to prioritize the allocation of interventions to high-risk home-dwelling patients to reduce readmissions, there are limitations that must be acknowledged. First, as with all observational studies there is concern for confounding and missing data. However, we included only patients with complete data and performed multivariable regression to account for known confounders. We were unable to account for patients’ origin (home, SNF, etc.) or those with multiple admissions prior to or following the study period. While these could be potential confounders, we have no reason to believe there was differential utilization of healthcare resources between groups. Next, we recognize the inability to eliminate risk of readmission to another facility. To mitigate this concern, we excluded patients who resided ≥25 miles from the hospital, as these individuals are likely to utilize a different medical center. The CCI was used to account for overall patient comorbidity. While this strategy is commonly employed when using observational data, patient disease states used to calculate the score relied heavily on the accuracy of ICD-10-CM coding. Regardless of these limitations, the study provides evidence that nearly 20% of patients are discharged with a medication regimen that places them in a high-risk category. Moreover, the effect size for 30-day readmission in the discharge to home subgroup was large, warranting further evaluation in prospective studies.

## 5. Conclusions

In brief, a MRS ≥ 20 was associated with an 80% higher risk of readmission in patients who are discharged home. The relatively small proportion of patients who fit this description could help identify those at highest risk of medication-related iatrogenesis and therefore effectively allocate limited healthcare resources in a standardized manner. Future studies will seek to confirm the hypothesis that a reduction in medication risk score will lead to reduced readmissions.

## Figures and Tables

**Table 1:** Clinical characteristics of all patients and subgroups.

Characteristic No. (%)		Overall	Subgroups		
		All Patients (*n* = 1386)	Discharged to Home (*n* = 680)	Discharged to Facility (*n* = 706)	*p*
Age *, years	65–74	358 (26)	239 (35)	119 (17)	<0.001
	≥75	783 (74)	441 (65)	587 (83)	<0.001
	Mean (SD)	80 (7)	78 (7)	82 (7)	<0.001
Sex	Female	784 (57)	362 (53)	422 (60)	0.009
Race	White	1168 (84)	552 (81)	616 (88)	<0.001
	Black	93 (7)	51 (8)	42 (6)	0.144
	Asian/Indian	84 (6)	53 (8)	31 (4)	0.002
	Other	41 (3)	24 (4)	40 (2)	0.027
Ethnicity	Hispanic	80 (6)	40 (6)	40 (6)	>0.99
BMI	kg/m^2^, mean (SD)	27.9 (7)	28.6 (7)	27 (7)	>0.99
Charlson Comorbidity Index	≥4	887 (64)	382 (56)	505 (72)	<0.001
Length of Stay	Days, mean (SD)	6 (4)	4 (3)	7 (5)	<0.001
Number of Medications	Mean (SD)	11 (5)	10 (5)	12 (5)	<0.001
Comorbidities	Heart Failure	454 (33)	209 (31)	245 (35)	0.114
	Diabetes Mellitus	449 (32)	224 (33)	225 (32)	0.691
	COPD	310 (22)	145 (21)	165 (23)	0.369
	Dementia	203 (15)	46 (7)	157 (22)	<0.001
	Myocardial Infarction	142 (10)	75 (11)	67 (9)	0.214
Readmission Within 30 Days	Readmission	194 (1)	85 (13)	109 (15)	0.284
	Time to Readmission, mean (SD), days	13.9 (7)	13 (9)	14 (9)	0.039
	By comorbidity: **				
	Heart Failure	75 (39)	30 (35)	45 (41)	0.394
	Diabetes Mellitus	70 (36)	33 (39)	37 (34)	0.472
	COPD	60 (31)	26 (31)	34 (31)	>0.99
	Dementia	24 (12)	4 (5)	20 (18)	0.006
	Myocardial Infarction	27 (14)	12 (14)	15 (14)	>0.99

SD = standard deviation; BMI = body mass index; COPD = chronic obstructive pulmonary disease; * = Age > 89 years censored; ** = Refers to readmitted patients who have been diagnosed with the listed disease state.

**Table 2:** Patient characteristics stratified by risk score and discharge disposition.

Characteristic, No. (%)		All Patients (*n* = 1386)			Discharged to Home (*n* = 680)			Discharged to Facility (*n* = 706)		
		MRS < 20 (*n* = 1157)	MRS ≥ 20 (*n* = 229)	*p*	MRS < 20 (*n* = 582)	MRS ≥ 20 (*n* = 98)	*p*	MRS < 20 (*n* = 575)	MRS ≥ 20 (*n* = 131)	*p*
Sex	Female	637 (55)	147 (64)	0.011	297 (51)	65 (66)	0.006	340 (59)	82 (63)	0.528
Age *	Years, mean (SD)	80 (7)	80 (7)	1	78 (7)	79 (7)	0.430	82 (6.8)	81 (7)	0.148
BMI	kg/m^2^, mean (SD),	27.7 (7)	28.9 (7)	0.018	28.5 (7)	29.4 (7)	0.239	26.8 (7)	28.6 (8)	0.021
CCI	>4	718 (62)	169 (74)	<0.001	317 (54)	65 (66)	0.027	401 (70)	104 (80)	0.032
Length of Stay	Days, mean (SD)	6 (5)	7 (5)	0.312	4 (3)	5 (3)	0.013	7 (5)	8 (5)	0.218
Number of Medications	5–6	247 (21)	2 (0.9)	<0.001	149 (26)	2 (2)	<0.001	98 (17)	0 (0)	<0.001
	7–14	750 (65)	71 (31)	<0.001	415 (61)	35 (36)	0.090	370 (64)	36 (27)	<0.001
	≥15	160 (14)	156 (68)	<0.001	53 (9)	61 (62)	<0.001	107 (19)	95 (73)	<0.001
	Mean (SD)	10 (14)	17 (6)	<0.001	9 (4)	17 (6)	<0.001	11 (4)	18 (6)	<0.001
Comorbidities	Heart Failure	368 (32)	86 (38)	0.078	169 (29)	40 (41)	0.017	199 (35)	46 (35)	>0.99
	Diabetes Mellitus	372 (32)	77 (34)	0.554	190 (33)	34 (35)	0.698	182 (32)	43 (33)	0.825
	COPD	248 (21)	62 (27)	0.045	117 (20)	28 (29)	0.044	131 (23)	34 (26)	0.465
	Dementia	156 (13)	47 (21)	0.002	34 (6)	12 (12)	<0.001	122 (21)	35 (27)	0.135
	Myocardial Infarction	120 (10)	22 (10)	>0.99	61 (10)	14 (14)	0.234	59 (10)	8 (6)	0.154
Readmission Within 30 days	Readmission	158 (13)	36 (16)	0.412	66 (10)	19 (19)	0.009	92 (16)	17 (13)	0.424
	Time to Readmission, mean (SD), days	14 (9)	12 (10)	0.221	14 (8)	12 (9)	0.17	14 (9)	14 (10)	0.387
	By comorbidity: **									
	Heart Failure	64 (41)	11 (31)	0.267	23 (35)	7 (37)	0.872	41 (45)	4 (24)	0.107
	Diabetes Mellitus	56 (35)	14 (39)	0.651	25 (38)	8 (42)	0.753	31 (34)	6 (35)	0.936
	COPD	47 (30)	13 (36)	0.483	20 (30)	6 (32)	0.867	27 (29)	7 (41)	0.325
	Dementia	19 (12)	5 (14)	0.742	3 (5)	1 (5)	>0.99	16 (17)	4 (24)	0.491
	Myocardial Infarction	23 (15)	4 (11)	0.536	9 (14)	3 (16)	0.827	14 (15)	1 (6)	<0.001

MRS = medication risk score; SD = standard deviation; BMI = body mass index; COPD = chronic obstructive pulmonary disease; BMI= Body mass index;* = Age > 89 years censored; ** = Refers to readmitted patients who have been diagnosed with the listed disease state.

**Table 3:** Multivariable Analysis of 30-day Readmission in All Patients and Subgroups.

	Parameter	Univariate		Multivariable	
		OR (95% CI)	*p*	OR (95% CI)	*p*
All Patients	MRS ≥ 20	1.18 (0.8–1.75)	0.411	1.06 (0.71–1.58)	0.787
	Age	1.00 (0.78–1.02)	0.846		
	Sex (male)	0.87 (0.64–1.18)	0.370		
	BMI ≤ 24.9	1.52 (1.12–2.06)	0.008		
	Length of Stay	1.07 (1.04–1.10)	<0.001	1.06 (1.03–1.09)	<0.001
	CCI > 4	2.25 (1.57–3.23)	<0.001	2.14 (1.48–3.08)	0.001
	Heart Failure	1.35 (0.99–1.85)	0.059		
	Diabetes	1.21 (0.88–1.68)	0.237		
	CKD Stage 3 or Worse	1.62 (1.18–2.22)	<0.001		
	MELD 20+	2.14 (0.75–6.12)	0.157		
Discharged Home	MRS ≥ 20	1.88 (1.07–3.30)	0.028	1.81 (1.02–3.21)	0.042
	Age	1.01 (0.98–1.04)	0.533		
	Sex (male)	1.23 (0.78–1.94)	0.384		
	BMI ≤ 24.9	1.65 (1.04–2.63)	0.034	1.72 (1.07–2.75)	0.024
	Length of Stay	1.01 (0.94–1.09)	0.752		
	CCI > 4	2.04 (1.24–3.33)	0.005	1.99 (1.21–3.27)	0.007
	Heart Failure	1.27 (0.79–2.05)	0.331		
	Diabetes	1.34 (0.84–2.15)	0.219		
	CKD Stage 3 or Worse	1.25 (0.76–2.06)	0.377		
	MELD 20+	1.33 (0.24–7.34)	0.741		
Discharged to a Facility	MRS ≥ 20	0.78 (0.45–1.37)	0.388	0.67 (0.38–1.19)	0.171
	Age	0.99 (0.96–1.02)	0.372		
	Sex (male)	0.64 (0.42–0.96)	0.032		
	BMI ≤ 24.9	1.40 (0.93–2.10)	0.111		
	Length of Stay	1.08 (1.04–1.12)	<0.001	1.08 (1.04–1.12)	<0.001
	CCI > 4	2.41 (1.40–4.16)	0.002	2.20 (1.24–3.90)	0.007
	Heart Failure	1.40 (0.92–2.12)	0.118		
	Diabetes	1.12 (0.73–1.72)	0.613		
	CKD Stage 3 or Worse	1.91 (1.26–2.89)	0.002	1.55 (1.01–2.40)	0.047
	MELD 20+	3.14 (0.80–12.4)	0.102		

MRS = medication risk score; CCI = Charlson Comorbidity Index; CKD = chronic kidney disease; MELD = model for end-stage liver disease; BMI = Body mass index.

## Data Availability

Data available on request.

## References

[1] Healthcare Cost and Utilization Project Facts and Figures 2008: Statistics on Hospital-Based Care in the United States 2008. http://www.hcup-us.ahrq.gov/reports/factsandfigures/2008/section1_TOC.jsp.

[2] Medicare Compare. https://www.medicare.gov/care-compare/.

[3] Lum H.D., Studenski S.A., Degenholtz H.B., Hardy S.E. (2012). Early hospital readmission is a predictor of one-year mortality in community-dwelling older Medicare beneficiaries. J. Gen. Intern. Med..

[4] Oscanoa T.J., Lizaraso F., Carvajal A. (2017). Hospital admissions due to adverse drug reactions in the elderly. A meta-analysis. Eur. J. Clin. Pharmacol..

[5] Parameswaran N.N., Chalmers L., Connolly M., Bereznicki B.J., Peterson G.M., Curtain C., Castelino R.L., Bereznicki L.R. (2016). Prediction of Hospitalization due to Adverse Drug Reactions in Elderly Community-Dwelling Patients (The PADR-EC Score). PLoS ONE.

[6] Tangiisuran B., Scutt G., Stevenson J., Wright J., Onder G., Petrovic M., Davies G. (2014). Development and validation of a risk model for predicting adverse drug reactions in older people during hospital stay: Brighton Adverse Drug Reactions Risk (BADRI) model. PLoS ONE.

[7] Onder G., Petrovic M., Tangiisuran B., Meinardi M.C., Markito-Notenboom W.P., Somers A., van der Cammen T.J. (2010). Development and validation of a score to assess risk of adverse drug reactions among in-hospital patients 65 years or older: The GerontoNet ADR risk score. Arch. Intern. Med..

[8] Ehrt U., Broich K., Larsen J.P., Ballard C., Aarsland D. (2010). Use of drugs with anticholinergic effect and impact on cognition in Parkinson’s disease: A cohort study. J. Neurol. Neurosurg. Psychiatry.

[9] Ancelin M.L., Artero S., Portet F., Dupuy A.M., Touchon J., Ritchie K. (2006). Non-degenerative mild cognitive impairment in elderly people and use of anticholinergic drugs: Longitudinal cohort study. BMJ.

[10] Carnahan R.M., Lund B.C., Perry P.J., Pollock B.G., Culp K.R. (2006). The Anticholinergic Drug Scale as a measure of drug-related anticholinergic burden: Associations with serum anticholinergic activity. J. Clin. Pharmacol..

[11] Soto R., Yaldou B. (2015). The Michigan Opioid Safety Score (MOSS): A Patient Safety and Nurse Empowerment Tool. J. Perianesth. Nurs..

[12] Webster L.R., Webster R.M. (2005). Predicting aberrant behaviors in opioid-treated patients: Preliminary validation of the Opioid Risk Tool. Pain Med..

[13] Cicali B.M.V., Knowlton C.H., Turgeon J. (2018). Application of a novel medication-related risk stratification strategy to a self-funded employer population. Benefits Q..

[14] Turgeon J., Michaud V., Stephen L.E., Badea G. (2017). Treatment Methods Having Reduced Drug-Related Toxicity and Methods of Identifying the Likelihood of Patient Harm from Prescribed Medications. U.S. Patent.

[15] Turgeon J., Michaud V., Cicali B. (2019). Population-Based Medication Risk Stratification and Personalized Medication Risk Score. U.S. Patent.

[16] Ratigan A.R., Michaud V., Turgeon J., Bikmetov R., Gaona V.G., Anderson H.D., Pulver G., Pace W.D. (2021). Longitudinal Association of a Medication Risk Score With Mortality Among Ambulatory Patients Acquired Through Electronic Health Record Data. J. Patient Saf..

[17] Bankes D.L., Jin H., Finnel S., Michaud V., Knowlton C.H., Turgeon J., Stein A. (2020). Association of a Novel Medication Risk Score with Adverse Drug Events and Other Pertinent Outcomes Among Participants of the Programs of All-Inclusive Care for the Elderly. Pharmacy.

[18] Charlson M.E., Pompei P., Ales K.L., MacKenzie C.R. (1987). A new method of classifying prognostic comorbidity in longitudinal studies: Development and validation. J. Chronic Dis..

[19] Quan H., Li B., Couris C.M., Fushimi K., Graham P., Hider P., Januel J., Sundararajan V. (2011). Updating and validating the Charlson comorbidity index and score for risk adjustment in hospital discharge abstracts using data from 6 countries. Am. J. Epidemiol..

[20] Zhang M., Holman C.D., Preen D.B., Brameld K. (2007). Repeat adverse drug reactions causing hospitalization in older Australians: A population-based longitudinal study 1980–2003. Br. J. Clin. Pharmacol..

[21] Requirements for Long Term Care Facilities, 483.45 Pharmacy Services. https://www.law.cornell.edu/cfr/text/42/483.45.

[22] Davies E.C.G., Mottram D.R., Howe P.H., Pirmohamed M. (2010). Emergency re-admissions to hospital due to adverse drug reactions within 1 year of the index admission. Br. J. Clin. Pharmacol..

[23] Requirements for Long Term Care Facilities, 483.21 Comprehensive Person-Centered Care Planning. https://www.law.cornell.edu/cfr/text/42/483.21.

[24] El Morabet N., Uitvlugt E.B., van den Bemt B.J.F., van den Bemt P., Janssen M.J.A., Karapinar-Carkit F. (2018). Prevalence and Preventability of Drug-Related Hospital Readmissions: A Systematic Review. J. Am. Geriatr. Soc..

